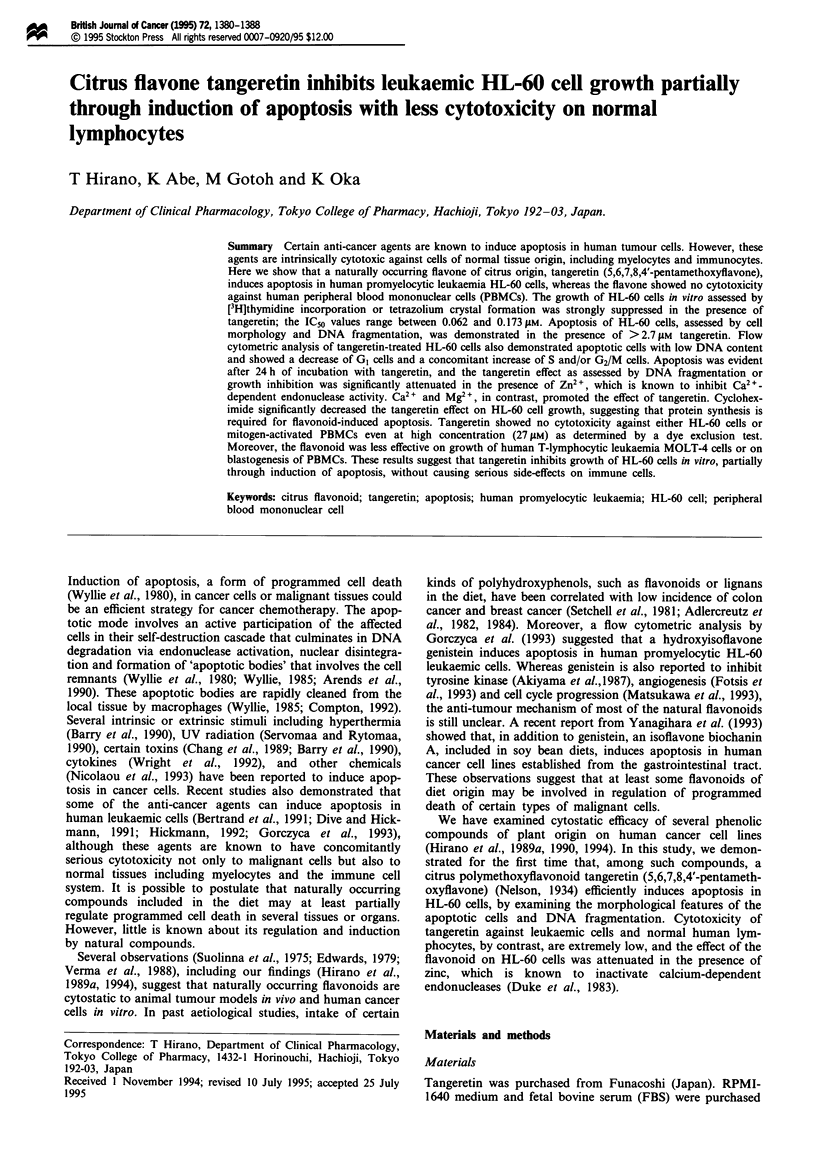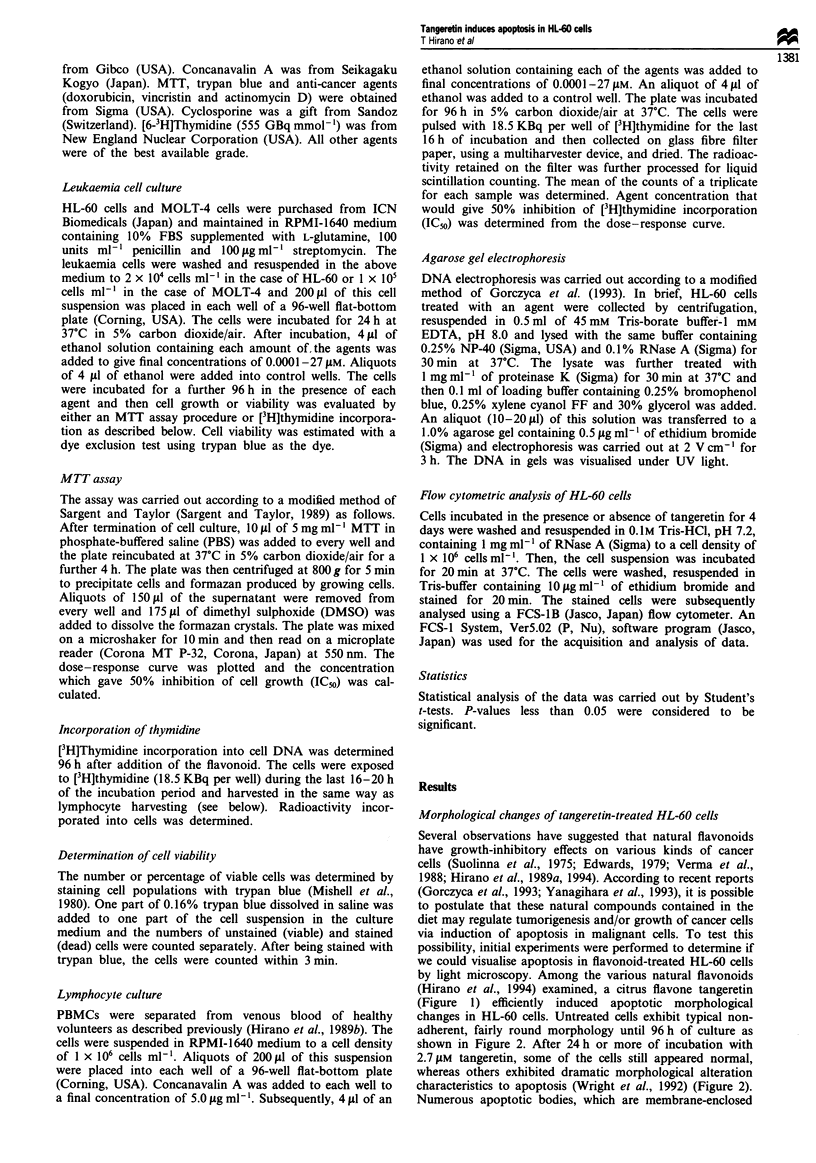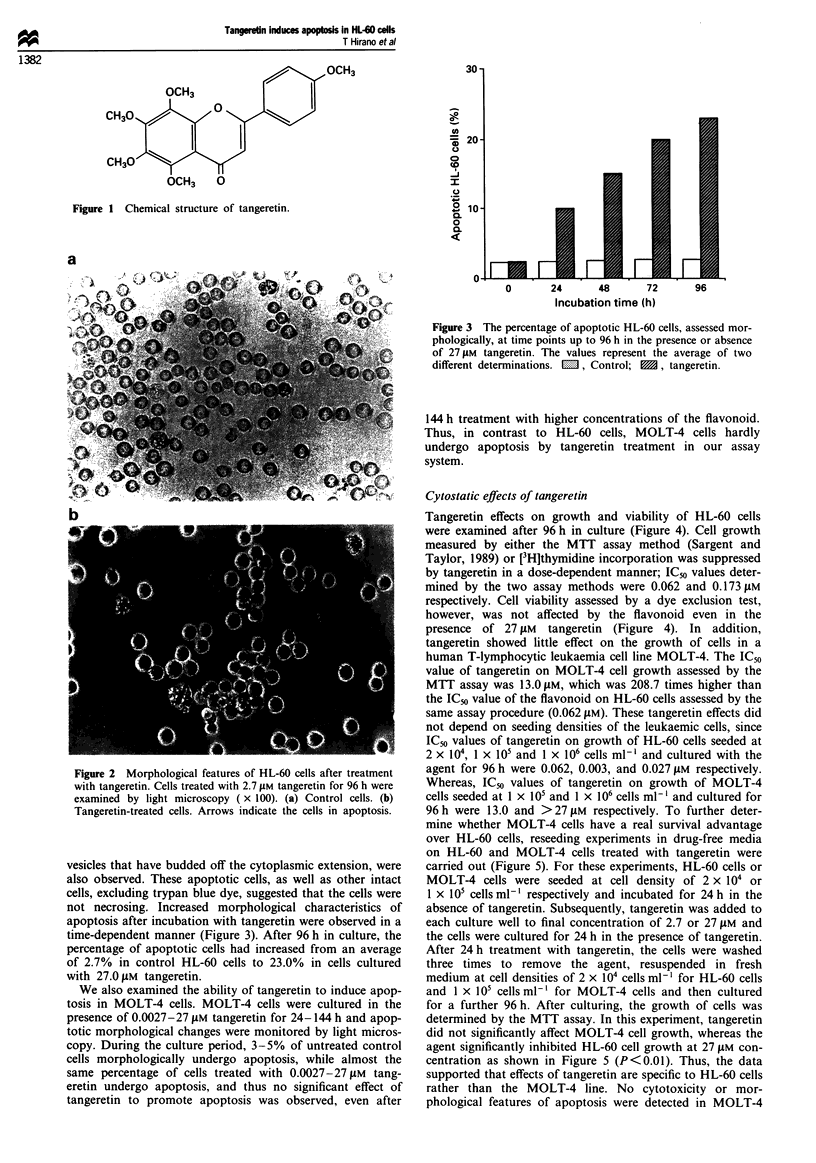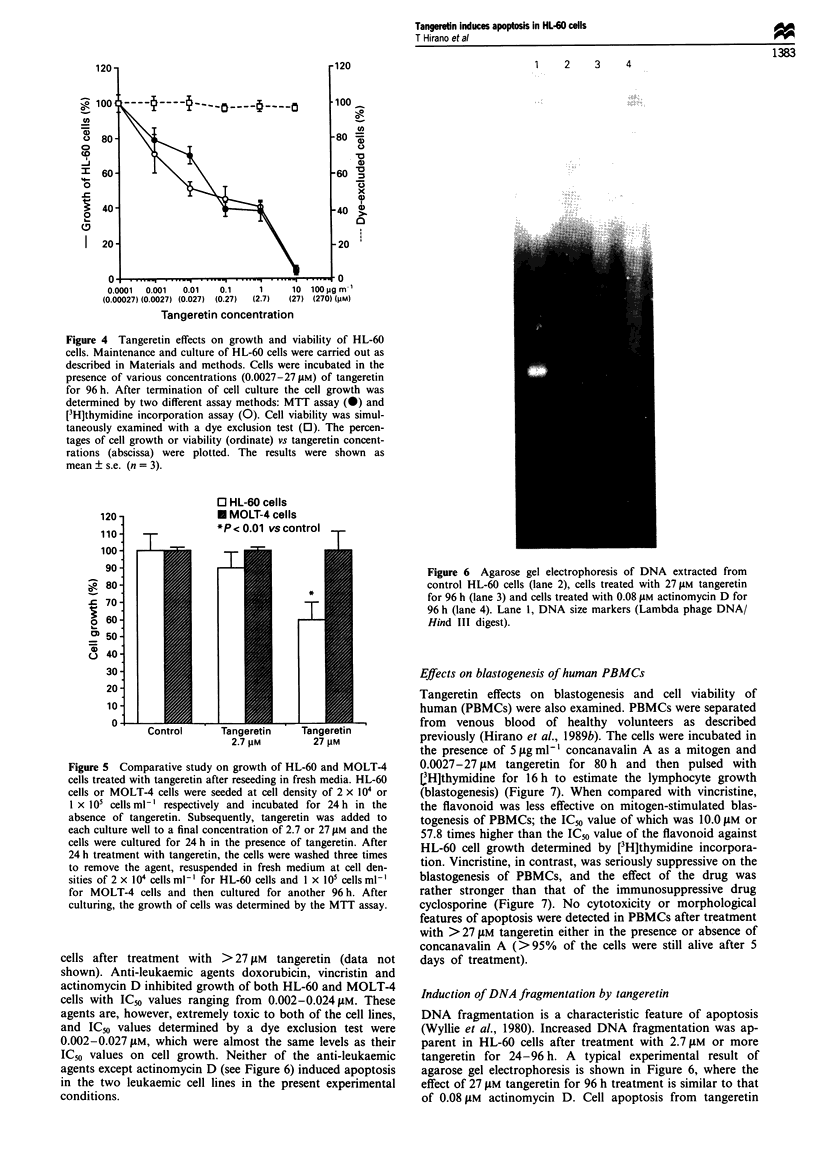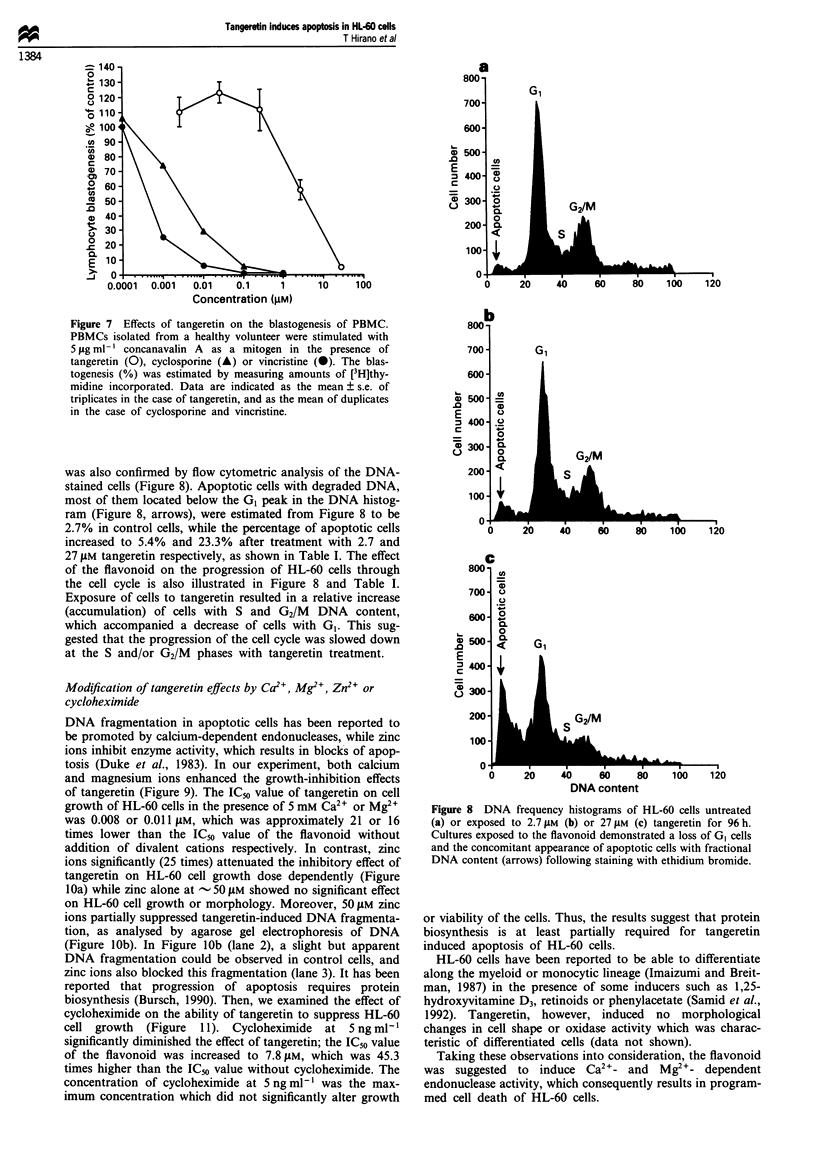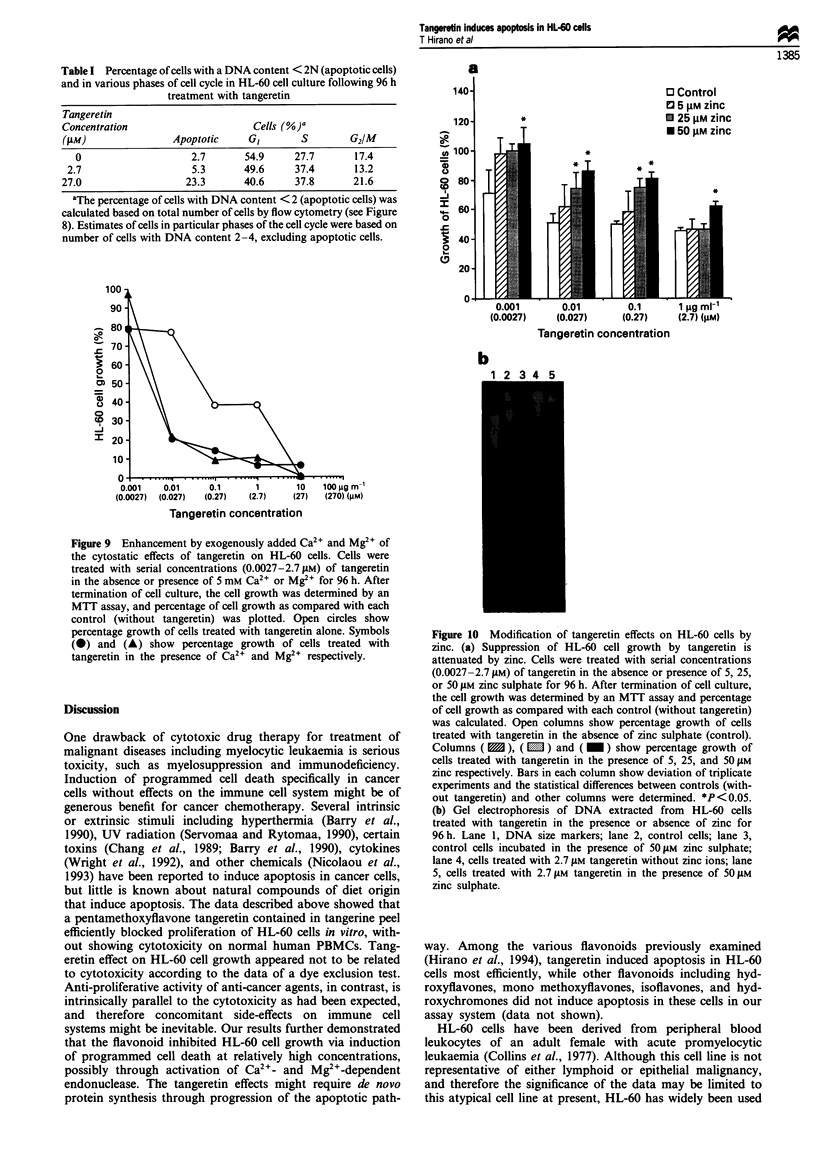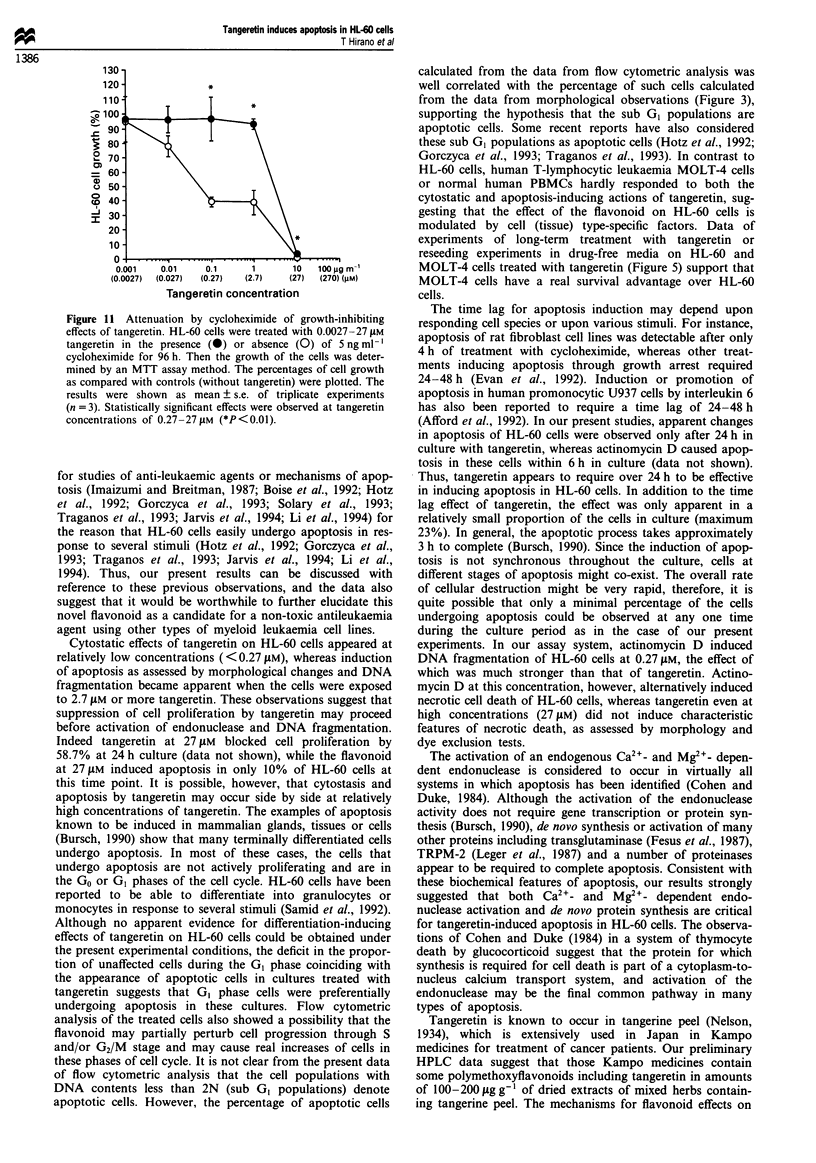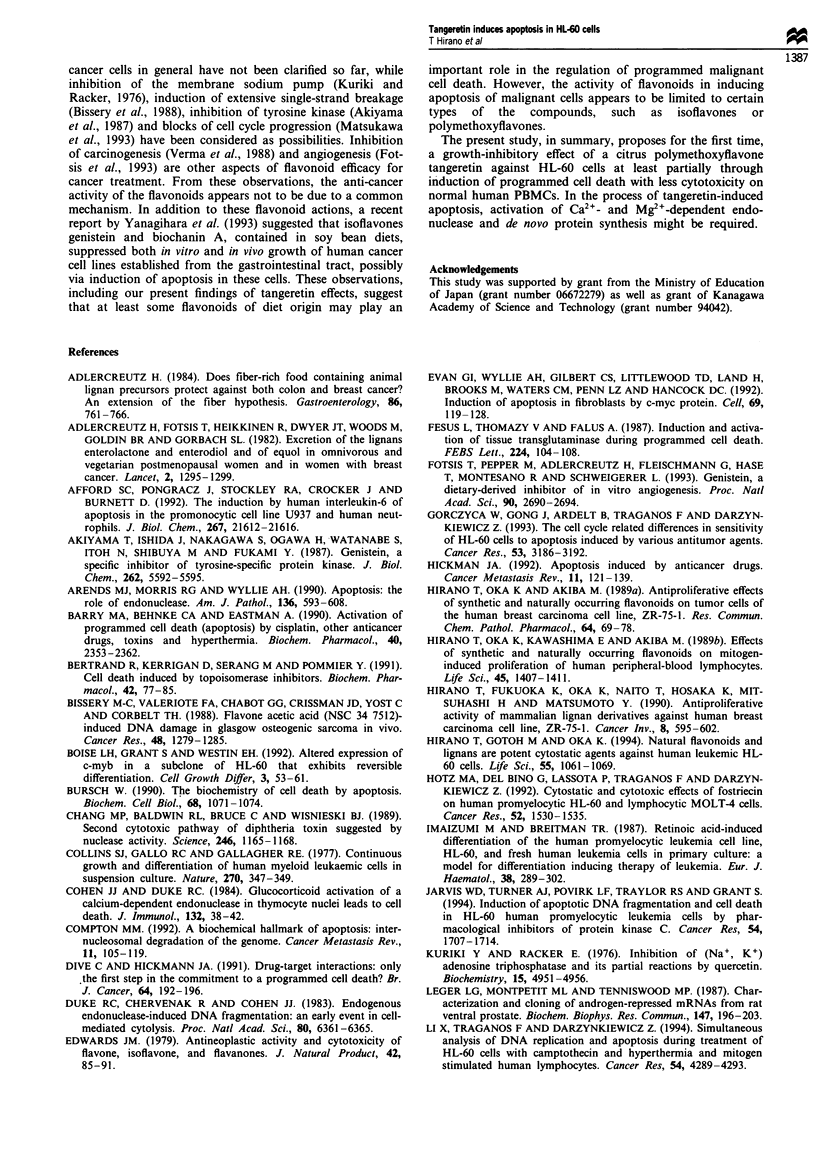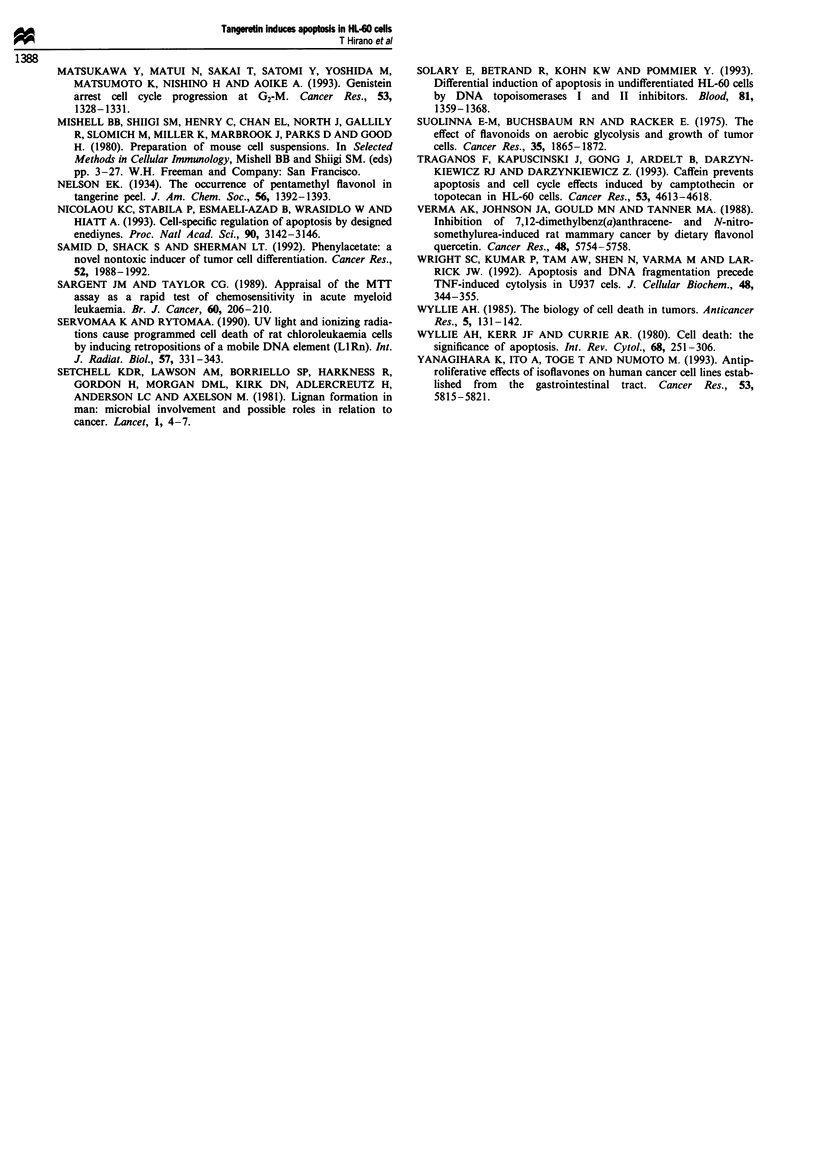# Citrus flavone tangeretin inhibits leukaemic HL-60 cell growth partially through induction of apoptosis with less cytotoxicity on normal lymphocytes.

**DOI:** 10.1038/bjc.1995.518

**Published:** 1995-12

**Authors:** T. Hirano, K. Abe, M. Gotoh, K. Oka

**Affiliations:** Department of Clinical Pharmacology, Tokyo College of Pharmacy, Japan.

## Abstract

**Images:**


British Journal of Cancer (1995) 72, 1380-1388
© 1995 Stockton Press All rights reserved 0007-0920/95 $12.00

### Citrus flavone tangeretin inhibits leukaemic HL-60 cell growth partially through induction of apoptosis with less cytotoxicity on normal lymphocytes

T Hirano, K Abe, M Gotoh and K Oka

*Department of Clinical Pharmacology, Tokyo College of Pharmacy, Hachioji, Tokyo 192-03, Japan.*

**Summary** Certain anti-cancer agents are known to induce apoptosis in human tumour cells. However, these agents are intrinsically cytotoxic against cells of normal tissue origin, including myelocytes and immunocytes. Here we show that a naturally occurring flavone of citrus origin, tangeretin (5,6,7,8,4'-pentamethoxyflavone), induces apoptosis in human promyelocytic leukaemia HL-60 cells, whereas the flavone showed no cytotoxicity against human peripheral blood mononuclear cells (PBMCs). The growth of HL-60 cells *in vitro* assessed by [$^3$H]thymidine incorporation or tetrazolium crystal formation was strongly suppressed in the presence of tangeretin; the $IC_{50}$ values range between 0.062 and 0.173 $\mu$M. Apoptosis of HL-60 cells, assessed by cell morphology and DNA fragmentation, was demonstrated in the presence of >2.7 $\mu$M tangeretin. Flow cytometric analysis of tangeretin-treated HL-60 cells also demonstrated apoptotic cells with low DNA content and showed a decrease of $G_1$ cells and a concomitant increase of S and/or $G_2$/M cells. Apoptosis was evident after 24 h of incubation with tangeretin, and the tangeretin effect as assessed by DNA fragmentation or growth inhibition was significantly attenuated in the presence of $Zn^{2+}$, which is known to inhibit $Ca^{2+}$-dependent endonuclease activity. $Ca^{2+}$ and $Mg^{2+}$, in contrast, promoted the effect of tangeretin. Cycloheximide significantly decreased the tangeretin effect on HL-60 cell growth, suggesting that protein synthesis is required for flavonoid-induced apoptosis. Tangeretin showed no cytotoxicity against either HL-60 cells or mitogen-activated PBMCs even at high concentration (27 $\mu$M) as determined by a dye exclusion test. Moreover, the flavonoid was less effective on growth of human T-lymphocytic leukaemia MOLT-4 cells or on blastogenesis of PBMCs. These results suggest that tangeretin inhibits growth of HL-60 cells *in vitro*, partially through induction of apoptosis, without causing serious side-effects on immune cells.

**Keywords:** citrus flavonoid; tangeretin; apoptosis; human promyelocytic leukaemia; HL-60 cell; peripheral blood mononuclear cell

Induction of apoptosis, a form of programmed cell death (Wyllie *et al.*, 1980), in cancer cells or malignant tissues could be an efficient strategy for cancer chemotherapy. The apoptotic mode involves an active participation of the affected cells in their self-destruction cascade that culminates in DNA degradation via endonuclease activation, nuclear disintegration and formation of 'apoptotic bodies' that involves the cell remnants (Wyllie *et al.*, 1980; Wyllie, 1985; Arends *et al.*, 1990). These apoptotic bodies are rapidly cleaned from the local tissue by macrophages (Wyllie, 1985; Compton, 1992). Several intrinsic or extrinsic stimuli including hyperthermia (Barry *et al.*, 1990), UV radiation (Servomaa and Rytomaa, 1990), certain toxins (Chang *et al.*, 1989; Barry *et al.*, 1990), cytokines (Wright *et al.*, 1992), and other chemicals (Nicolaou *et al.*, 1993) have been reported to induce apoptosis in cancer cells. Recent studies also demonstrated that some of the anti-cancer agents can induce apoptosis in human leukaemic cells (Bertrand *et al.*, 1991; Dive and Hickmann, 1991; Hickmann, 1992; Gorczyca *et al.*, 1993), although these agents are known to have concomitantly serious cytotoxicity not only to malignant cells but also to normal tissues including myelocytes and the immune cell system. It is possible to postulate that naturally occurring compounds included in the diet may at least partially regulate programmed cell death in several tissues or organs. However, little is known about its regulation and induction by natural compounds.

Several observations (Suolinna *et al.*, 1975; Edwards, 1979; Verma *et al.*, 1988), including our findings (Hirano *et al.*, 1989*a*, 1994), suggest that naturally occurring flavonoids are cytostatic to animal tumour models *in vivo* and human cancer cells *in vitro*. In past aetiological studies, intake of certain kinds of polyhydroxyphenols, such as flavonoids or lignans in the diet, have been correlated with low incidence of colon cancer and breast cancer (Setchell *et al.*, 1981; Adlercreutz *et al.*, 1982, 1984). Moreover, a flow cytometric analysis by Gorczyca *et al.* (1993) suggested that a hydroxyisoflavone genistein induces apoptosis in human promyelocytic HL-60 leukaemic cells. Whereas genistein is also reported to inhibit tyrosine kinase (Akiyama *et al.*,1987), angiogenesis (Fotsis *et al.*, 1993) and cell cycle progression (Matsukawa *et al.*, 1993), the anti-tumour mechanism of most of the natural flavonoids is still unclear. A recent report from Yanagihara *et al.* (1993) showed that, in addition to genistein, an isoflavone biochanin A, included in soy bean diets, induces apoptosis in human cancer cell lines established from the gastrointestinal tract. These observations suggest that at least some flavonoids of diet origin may be involved in regulation of programmed death of certain types of malignant cells.

We have examined cytostatic efficacy of several phenolic compounds of plant origin on human cancer cell lines (Hirano *et al.*, 1989*a*, 1990, 1994). In this study, we demonstrated for the first time that, among such compounds, a citrus polymethoxyflavonoid tangeretin (5,6,7,8,4'-pentamethoxyflavone) (Nelson, 1934) efficiently induces apoptosis in HL-60 cells, by examining the morphological features of the apoptotic cells and DNA fragmentation. Cytotoxicity of tangeretin against leukaemic cells and normal human lymphocytes, by contrast, are extremely low, and the effect of the flavonoid on HL-60 cells was attenuated in the presence of zinc, which is known to inactivate calcium-dependent endonucleases (Duke *et al.*, 1983).

Correspondence: T Hirano, Department of Clinical Pharmacology, Tokyo College of Pharmacy, 1432-1 Horinouchi, Hachioji, Tokyo 192-03, Japan
Received 1 November 1994; revised 10 July 1995; accepted 25 July 1995

### Materials and methods

### *Materials*

Tangeretin was purchased from Funacoshi (Japan). RPMI-1640 medium and fetal bovine serum (FBS) were purchased from Gibco (USA). Concanavalin A was from Seikagaku Kogyo (Japan). MTT, trypan blue and anti-cancer agents (doxorubicin, vincristin and actinomycin D) were obtained from Sigma (USA). Cyclosporine was a gift from Sandoz (Switzerland). [6-$^3$H]Thymidine (555 GBq mmol$^{-1}$) was from New England Nuclear Corporation (USA). All other agents were of the best available grade.

*Leukaemia cell culture*

HL-60 cells and MOLT-4 cells were purchased from ICN Biomedicals (Japan) and maintained in RPMI-1640 medium containing 10% FBS supplemented with L-glutamine, 100 units ml$^{-1}$ penicillin and 100 μg ml$^{-1}$ streptomycin. The leukaemia cells were washed and resuspended in the above medium to $2 \times 10^4$ cells ml$^{-1}$ in the case of HL-60 or $1 \times 10^5$ cells ml$^{-1}$ in the case of MOLT-4 and 200 μl of this cell suspension was placed in each well of a 96-well flat-bottom plate (Corning, USA). The cells were incubated for 24 h at 37°C in 5% carbon dioxide/air. After incubation, 4 μl of ethanol solution containing each amount of the agents was added to give final concentrations of 0.0001–27 μM. Aliquots of 4 μl of ethanol were added into control wells. The cells were incubated for a further 96 h in the presence of each agent and then cell growth or viability was evaluated by either an MTT assay procedure or [$^3$H]thymidine incorporation as described below. Cell viability was estimated with a dye exclusion test using trypan blue as the dye.

*MTT assay*

The assay was carried out according to a modified method of Sargent and Taylor (Sargent and Taylor, 1989) as follows. After termination of cell culture, 10 μl of 5 mg ml$^{-1}$ MTT in phosphate-buffered saline (PBS) was added to every well and the plate reincubated at 37°C in 5% carbon dioxide/air for a further 4 h. The plate was then centrifuged at 800 *g* for 5 min to precipitate cells and formazan produced by growing cells. Aliquots of 150 μl of the supernatant were removed from every well and 175 μl of dimethyl sulphoxide (DMSO) was added to dissolve the formazan crystals. The plate was mixed on a microshaker for 10 min and then read on a microplate reader (Corona MT P-32, Corona, Japan) at 550 nm. The dose–response curve was plotted and the concentration which gave 50% inhibition of cell growth ($IC_{50}$) was calculated.

*Incorporation of thymidine*

[$^3$H]Thymidine incorporation into cell DNA was determined 96 h after addition of the flavonoid. The cells were exposed to [$^3$H]thymidine (18.5 KBq per well) during the last 16–20 h of the incubation period and harvested in the same way as lymphocyte harvesting (see below). Radioactivity incorporated into cells was determined.

*Determination of cell viability*

The number or percentage of viable cells was determined by staining cell populations with trypan blue (Mishell *et al.*, 1980). One part of 0.16% trypan blue dissolved in saline was added to one part of the cell suspension in the culture medium and the numbers of unstained (viable) and stained (dead) cells were counted separately. After being stained with trypan blue, the cells were counted within 3 min.

*Lymphocyte culture*

PBMCs were separated from venous blood of healthy volunteers as described previously (Hirano *et al.*, 1989*b*). The cells were suspended in RPMI-1640 medium to a cell density of $1 \times 10^6$ cells ml$^{-1}$. Aliquots of 200 μl of this suspension were placed into each well of a 96-well flat-bottom plate (Corning, USA). Concanavalin A was added to each well to a final concentration of 5.0 μg ml$^{-1}$. Subsequently, 4 μl of an ethanol solution containing each of the agents was added to final concentrations of 0.0001–27 μM. An aliquot of 4 μl of ethanol was added to a control well. The plate was incubated for 96 h in 5% carbon dioxide/air at 37°C. The cells were pulsed with 18.5 KBq per well of [$^3$H]thymidine for the last 16 h of incubation and then collected on glass fibre filter paper, using a multiharvester device, and dried. The radioactivity retained on the filter was further processed for liquid scintillation counting. The mean of the counts of a triplicate for each sample was determined. Agent concentration that would give 50% inhibition of [$^3$H]thymidine incorporation ($IC_{50}$) was determined from the dose–response curve.

*Agarose gel electrophoresis*

DNA electrophoresis was carried out according to a modified method of Gorczyca *et al.* (1993). In brief, HL-60 cells treated with an agent were collected by centrifugation, resuspended in 0.5 ml of 45 mM Tris-borate buffer-1 mM EDTA, pH 8.0 and lysed with the same buffer containing 0.25% NP-40 (Sigma, USA) and 0.1% RNase A (Sigma) for 30 min at 37°C. The lysate was further treated with 1 mg ml$^{-1}$ of proteinase K (Sigma) for 30 min at 37°C and then 0.1 ml of loading buffer containing 0.25% bromophenol blue, 0.25% xylene cyanol FF and 30% glycerol was added. An aliquot (10–20 μl) of this solution was transferred to a 1.0% agarose gel containing 0.5 μg ml$^{-1}$ of ethidium bromide (Sigma) and electrophoresis was carried out at 2 V cm$^{-1}$ for 3 h. The DNA in gels was visualised under UV light.

*Flow cytometric analysis of HL-60 cells*

Cells incubated in the presence or absence of tangeretin for 4 days were washed and resuspended in 0.1M Tris-HCl, pH 7.2, containing 1 mg ml$^{-1}$ of RNase A (Sigma) to a cell density of $1 \times 10^6$ cells ml$^{-1}$. Then, the cell suspension was incubated for 20 min at 37°C. The cells were washed, resuspended in Tris-buffer containing 10 μg ml$^{-1}$ of ethidium bromide and stained for 20 min. The stained cells were subsequently analysed using a FCS-1B (Jasco, Japan) flow cytometer. An FCS-1 System, Ver5.02 (P, Nu), software program (Jasco, Japan) was used for the acquisition and analysis of data.

*Statistics*

Statistical analysis of the data was carried out by Student's *t*-tests. *P*-values less than 0.05 were considered to be significant.

### Results

*Morphological changes of tangeretin-treated HL-60 cells*

Several observations have suggested that natural flavonoids have growth-inhibitory effects on various kinds of cancer cells (Suolinna *et al.*, 1975; Edwards, 1979; Verma *et al.*, 1988; Hirano *et al.*, 1989*a*, 1994). According to recent reports (Gorczyca *et al.*, 1993; Yanagihara *et al.*, 1993), it is possible to postulate that these natural compounds contained in the diet may regulate tumorigenesis and/or growth of cancer cells via induction of apoptosis in malignant cells. To test this possibility, initial experiments were performed to determine if we could visualise apoptosis in flavonoid-treated HL-60 cells by light microscopy. Among the various natural flavonoids (Hirano *et al.*, 1994) examined, a citrus flavone tangeretin (Figure 1) efficiently induced apoptotic morphological changes in HL-60 cells. Untreated cells exhibit typical non-adherent, fairly round morphology until 96 h of culture as shown in Figure 2. After 24 h or more of incubation with 2.7 μM tangeretin, some of the cells still appeared normal, whereas others exhibited dramatic morphological alteration characteristics to apoptosis (Wright *et al.*, 1992) (Figure 2). Numerous apoptotic bodies, which are membrane-enclosed

**Figure 1** Chemical structure of tangeretin.

**Figure 2** Morphological features of HL-60 cells after treatment with tangeretin. Cells treated with 2.7 $\mu$M tangeretin for 96 h were examined by light microscopy ( × 100). (**a**) Control cells. (**b**) Tangeretin-treated cells. Arrows indicate the cells in apoptosis.

vesicles that have budded off the cytoplasmic extension, were also observed. These apoptotic cells, as well as other intact cells, excluding trypan blue dye, suggested that the cells were not necrosing. Increased morphological characteristics of apoptosis after incubation with tangeretin were observed in a time-dependent manner (Figure 3). After 96 h in culture, the percentage of apoptotic cells had increased from an average of 2.7% in control HL-60 cells to 23.0% in cells cultured with 27.0 $\mu$M tangeretin.

We also examined the ability of tangeretin to induce apoptosis in MOLT-4 cells. MOLT-4 cells were cultured in the presence of 0.0027–27 $\mu$M tangeretin for 24–144 h and apoptotic morphological changes were monitored by light microscopy. During the culture period, 3–5% of untreated control cells morphologically undergo apoptosis, while almost the same percentage of cells treated with 0.0027–27 $\mu$M tangeretin undergo apoptosis, and thus no significant effect of tangeretin to promote apoptosis was observed, even after

**Figure 3** The percentage of apoptotic HL-60 cells, assessed morphologically, at time points up to 96 h in the presence or absence of 27 $\mu$M tangeretin. The values represent the average of two different determinations. ▒, Control; ▨, tangeretin.

144 h treatment with higher concentrations of the flavonoid. Thus, in contrast to HL-60 cells, MOLT-4 cells hardly undergo apoptosis by tangeretin treatment in our assay system.

*Cytostatic effects of tangeretin*

Tangeretin effects on growth and viability of HL-60 cells were examined after 96 h in culture (Figure 4). Cell growth measured by either the MTT assay method (Sargent and Taylor, 1989) or [$^3$H]thymidine incorporation was suppressed by tangeretin in a dose-dependent manner; $IC_{50}$ values determined by the two assay methods were 0.062 and 0.173 $\mu$M respectively. Cell viability assessed by a dye exclusion test, however, was not affected by the flavonoid even in the presence of 27 $\mu$M tangeretin (Figure 4). In addition, tangeretin showed little effect on the growth of cells in a human T-lymphocytic leukaemia cell line MOLT-4. The $IC_{50}$ value of tangeretin on MOLT-4 cell growth assessed by the MTT assay was 13.0 $\mu$M, which was 208.7 times higher than the $IC_{50}$ value of the flavonoid on HL-60 cells assessed by the same assay procedure (0.062 $\mu$M). These tangeretin effects did not depend on seeding densities of the leukaemic cells, since $IC_{50}$ values of tangeretin on growth of HL-60 cells seeded at $2 \times 10^4$, $1 \times 10^5$ and $1 \times 10^6$ cells ml$^{-1}$ and cultured with the agent for 96 h were 0.062, 0.003, and 0.027 $\mu$M respectively. Whereas, $IC_{50}$ values of tangeretin on growth of MOLT-4 cells seeded at $1 \times 10^5$ and $1 \times 10^6$ cells ml$^{-1}$ and cultured for 96 h were 13.0 and $>$27 $\mu$M respectively. To further determine whether MOLT-4 cells have a real survival advantage over HL-60 cells, reseeding experiments in drug-free media on HL-60 and MOLT-4 cells treated with tangeretin were carried out (Figure 5). For these experiments, HL-60 cells or MOLT-4 cells were seeded at cell density of $2 \times 10^4$ or $1 \times 10^5$ cells ml$^{-1}$ respectively and incubated for 24 h in the absence of tangeretin. Subsequently, tangeretin was added to each culture well to final concentration of 2.7 or 27 $\mu$M and the cells were cultured for 24 h in the presence of tangeretin. After 24 h treatment with tangeretin, the cells were washed three times to remove the agent, resuspended in fresh medium at cell densities of $2 \times 10^4$ cells ml$^{-1}$ for HL-60 cells and $1 \times 10^5$ cells ml$^{-1}$ for MOLT-4 cells and then cultured for a further 96 h. After culturing, the growth of cells was determined by the MTT assay. In this experiment, tangeretin did not significantly affect MOLT-4 cell growth, whereas the agent significantly inhibited HL-60 cell growth at 27 $\mu$M concentration as shown in Figure 5 ($P<0.01$). Thus, the data supported that effects of tangeretin are specific to HL-60 cells rather than the MOLT-4 line. No cytotoxicity or morphological features of apoptosis were detected in MOLT-4

**Figure 4** Tangeretin effects on growth and viability of HL-60 cells. Maintenance and culture of HL-60 cells were carried out as described in Materials and methods. Cells were incubated in the presence of various concentrations (0.0027–27 μM) of tangeretin for 96 h. After termination of cell culture the cell growth was determined by two different assay methods: MTT assay (●) and [$^3$H]thymidine incorporation assay (O). Cell viability was simultaneously examined with a dye exclusion test (□). The percentages of cell growth or viability (ordinate) *vs* tangeretin concentrations (abscissa) were plotted. The results were shown as mean ± s.e. ($n = 3$).

**Figure 5** Comparative study on growth of HL-60 and MOLT-4 cells treated with tangeretin after reseeding in fresh media. HL-60 cells or MOLT-4 cells were seeded at cell density of $2 \times 10^4$ or $1 \times 10^5$ cells ml$^{-1}$ respectively and incubated for 24 h in the absence of tangeretin. Subsequently, tangeretin was added to each culture well to a final concentration of 2.7 or 27 μM and the cells were cultured for 24 h in the presence of tangeretin. After 24 h treatment with tangeretin, the cells were washed three times to remove the agent, resuspended in fresh medium at cell densities of $2 \times 10^4$ cells ml$^{-1}$ for HL-60 cells and $1 \times 10^5$ cells ml$^{-1}$ for MOLT-4 cells and then cultured for another 96 h. After culturing, the growth of cells was determined by the MTT assay.

cells after treatment with >27 μM tangeretin (data not shown). Anti-leukaemic agents doxorubicin, vincristin and actinomycin D inhibited growth of both HL-60 and MOLT-4 cells with $IC_{50}$ values ranging from 0.002–0.024 μM. These agents are, however, extremely toxic to both of the cell lines, and $IC_{50}$ values determined by a dye exclusion test were 0.002–0.027 μM, which were almost the same levels as their $IC_{50}$ values on cell growth. Neither of the anti-leukaemic agents except actinomycin D (see Figure 6) induced apoptosis in the two leukaemic cell lines in the present experimental conditions.

**Figure 6** Agarose gel electrophoresis of DNA extracted from control HL-60 cells (lane 2), cells treated with 27 μM tangeretin for 96 h (lane 3) and cells treated with 0.08 μM actinomycin D for 96 h (lane 4). Lane 1, DNA size markers (Lambda phage DNA/*Hind* III digest).

### *Effects on blastogenesis of human PBMCs*

Tangeretin effects on blastogenesis and cell viability of human (PBMCs) were also examined. PBMCs were separated from venous blood of healthy volunteers as described previously (Hirano *et al.*, 1989*b*). The cells were incubated in the presence of 5 μg ml$^{-1}$ concanavalin A as a mitogen and 0.0027–27 μM tangeretin for 80 h and then pulsed with [$^3$H]thymidine for 16 h to estimate the lymphocyte growth (blastogenesis) (Figure 7). When compared with vincristine, the flavonoid was less effective on mitogen-stimulated blastogenesis of PBMCs; the $IC_{50}$ value of which was 10.0 μM or 57.8 times higher than the $IC_{50}$ value of the flavonoid against HL-60 cell growth determined by [$^3$H]thymidine incorporation. Vincristine, in contrast, was seriously suppressive on the blastogenesis of PBMCs, and the effect of the drug was rather stronger than that of the immunosuppressive drug cyclosporine (Figure 7). No cytotoxicity or morphological features of apoptosis were detected in PBMCs after treatment with >27 μM tangeretin either in the presence or absence of concanavalin A (>95% of the cells were still alive after 5 days of treatment).

### *Induction of DNA fragmentation by tangeretin*

DNA fragmentation is a characteristic feature of apoptosis (Wyllie *et al.*, 1980). Increased DNA fragmentation was apparent in HL-60 cells after treatment with 2.7 μM or more tangeretin for 24–96 h. A typical experimental result of agarose gel electrophoresis is shown in Figure 6, where the effect of 27 μM tangeretin for 96 h treatment is similar to that of 0.08 μM actinomycin D. Cell apoptosis from tangeretin

**Figure 7** Effects of tangeretin on the blastogenesis of PBMC. PBMCs isolated from a healthy volunteer were stimulated with 5 μg ml$^{-1}$ concanavalin A as a mitogen in the presence of tangeretin (○), cyclosporine (▲) or vincristine (●). The blastogenesis (%) was estimated by measuring amounts of [$^3$H]thymidine incorporated. Data are indicated as the mean ± s.e. of triplicates in the case of tangeretin, and as the mean of duplicates in the case of cyclosporine and vincristine.

**Figure 8** DNA frequency histograms of HL-60 cells untreated (**a**) or exposed to 2.7 μM (**b**) or 27 μM (**c**) tangeretin for 96 h. Cultures exposed to the flavonoid demonstrated a loss of $G_1$ cells and the concomitant appearance of apoptotic cells with fractional DNA content (arrows) following staining with ethidium bromide.

was also confirmed by flow cytometric analysis of the DNA-stained cells (Figure 8). Apoptotic cells with degraded DNA, most of them located below the $G_1$ peak in the DNA histogram (Figure 8, arrows), were estimated from Figure 8 to be 2.7% in control cells, while the percentage of apoptotic cells increased to 5.4% and 23.3% after treatment with 2.7 and 27 μM tangeretin respectively, as shown in Table I. The effect of the flavonoid on the progression of HL-60 cells through the cell cycle is also illustrated in Figure 8 and Table I. Exposure of cells to tangeretin resulted in a relative increase (accumulation) of cells with S and $G_2$/M DNA content, which accompanied a decrease of cells with $G_1$. This suggested that the progression of the cell cycle was slowed down at the S and/or $G_2$/M phases with tangeretin treatment.

### *Modification of tangeretin effects by $Ca^{2+}$, $Mg^{2+}$, $Zn^{2+}$ or cycloheximide*

DNA fragmentation in apoptotic cells has been reported to be promoted by calcium-dependent endonucleases, while zinc ions inhibit enzyme activity, which results in blocks of apoptosis (Duke *et al.*, 1983). In our experiment, both calcium and magnesium ions enhanced the growth-inhibition effects of tangeretin (Figure 9). The $IC_{50}$ value of tangeretin on cell growth of HL-60 cells in the presence of 5 mM $Ca^{2+}$ or $Mg^{2+}$ was 0.008 or 0.011 μM, which was approximately 21 or 16 times lower than the $IC_{50}$ value of the flavonoid without addition of divalent cations respectively. In contrast, zinc ions significantly (25 times) attenuated the inhibitory effect of tangeretin on HL-60 cell growth dose dependently (Figure 10a) while zinc alone at ~50 μM showed no significant effect on HL-60 cell growth or morphology. Moreover, 50 μM zinc ions partially suppressed tangeretin-induced DNA fragmentation, as analysed by agarose gel electrophoresis of DNA (Figure 10b). In Figure 10b (lane 2), a slight but apparent DNA fragmentation could be observed in control cells, and zinc ions also blocked this fragmentation (lane 3). It has been reported that progression of apoptosis requires protein biosynthesis (Bursch, 1990). Then, we examined the effect of cycloheximide on the ability of tangeretin to suppress HL-60 cell growth (Figure 11). Cycloheximide at 5 ng ml$^{-1}$ significantly diminished the effect of tangeretin; the $IC_{50}$ value of the flavonoid was increased to 7.8 μM, which was 45.3 times higher than the $IC_{50}$ value without cycloheximide. The concentration of cycloheximide at 5 ng ml$^{-1}$ was the maximum concentration which did not significantly alter growth or viability of the cells. Thus, the results suggest that protein biosynthesis is at least partially required for tangeretin induced apoptosis of HL-60 cells.

HL-60 cells have been reported to be able to differentiate along the myeloid or monocytic lineage (Imaizumi and Breitman, 1987) in the presence of some inducers such as 1,25-hydroxyvitamine $D_3$, retinoids or phenylacetate (Samid *et al.*, 1992). Tangeretin, however, induced no morphological changes in cell shape or oxidase activity which was characteristic of differentiated cells (data not shown).

Taking these observations into consideration, the flavonoid was suggested to induce $Ca^{2+}$- and $Mg^{2+}$- dependent endonuclease activity, which consequently results in programmed cell death of HL-60 cells.

**Table I** Percentage of cells with a DNA content <2N (apoptotic cells) and in various phases of cell cycle in HL-60 cell culture following 96 h treatment with tangeretin

| *Tangeretin Concentration (μM)* | *Apoptotic* | *Cells (%)[a]* $G_1$ | *S* | $G_2/M$ |
|---|---|---|---|---|
| 0 | 2.7 | 54.9 | 27.7 | 17.4 |
| 2.7 | 5.3 | 49.6 | 37.4 | 13.2 |
| 27.0 | 23.3 | 40.6 | 37.8 | 21.6 |

[a]The percentage of cells with DNA content <2 (apoptotic cells) was calculated based on total number of cells by flow cytometry (see Figure 8). Estimates of cells in particular phases of the cell cycle were based on number of cells with DNA content 2–4, excluding apoptotic cells.

**Figure 9** Enhancement by exogenously added $Ca^{2+}$ and $Mg^{2+}$ of the cytostatic effects of tangeretin on HL-60 cells. Cells were treated with serial concentrations (0.0027–2.7 μM) of tangeretin in the absence or presence of 5 mM $Ca^{2+}$ or $Mg^{2+}$ for 96 h. After termination of cell culture, the cell growth was determined by an MTT assay, and percentage of cell growth as compared with each control (without tangeretin) was plotted. Open circles show percentage growth of cells treated with tangeretin alone. Symbols (●) and (▲) show percentage growth of cells treated with tangeretin in the presence of $Ca^{2+}$ and $Mg^{2+}$ respectively.

**Figure 10** Modification of tangeretin effects on HL-60 cells by zinc. (**a**) Suppression of HL-60 cell growth by tangeretin is attenuated by zinc. Cells were treated with serial concentrations (0.0027–2.7 μM) of tangeretin in the absence or presence of 5, 25, or 50 μM zinc sulphate for 96 h. After termination of cell culture, the cell growth was determined by an MTT assay and percentage of cell growth as compared with each control (without tangeretin) was calculated. Open columns show percentage growth of cells treated with tangeretin in the absence of zinc sulphate (control). Columns (▨), (▩) and (■) show percentage growth of cells treated with tangeretin in the presence of 5, 25, and 50 μM zinc respectively. Bars in each column show deviation of triplicate experiments and the statistical differences between controls (without tangeretin) and other columns were determined. $*P<0.05$. (**b**) Gel electrophoresis of DNA extracted from HL-60 cells treated with tangeretin in the presence or absence of zinc for 96 h. Lane 1, DNA size markers; lane 2, control cells; lane 3, control cells incubated in the presence of 50 μM zinc sulphate; lane 4, cells treated with 2.7 μM tangeretin without zinc ions; lane 5, cells treated with 2.7 μM tangeretin in the presence of 50 μM zinc sulphate.

### Discussion

One drawback of cytotoxic drug therapy for treatment of malignant diseases including myelocytic leukaemia is serious toxicity, such as myelosuppression and immunodeficiency. Induction of programmed cell death specifically in cancer cells without effects on the immune cell system might be of generous benefit for cancer chemotherapy. Several intrinsic or extrinsic stimuli including hyperthermia (Barry *et al.*, 1990), UV radiation (Servomaa and Rytomaa, 1990), certain toxins (Chang *et al.*, 1989; Barry *et al.*, 1990), cytokines (Wright *et al.*, 1992), and other chemicals (Nicolaou *et al.*, 1993) have been reported to induce apoptosis in cancer cells, but little is known about natural compounds of diet origin that induce apoptosis. The data described above showed that a pentamethoxyflavone tangeretin contained in tangerine peel efficiently blocked proliferation of HL-60 cells *in vitro*, without showing cytotoxicity on normal human PBMCs. Tangeretin effect on HL-60 cell growth appeared not to be related to cytotoxicity according to the data of a dye exclusion test. Anti-proliferative activity of anti-cancer agents, in contrast, is intrinsically parallel to the cytotoxicity as had been expected, and therefore concomitant side-effects on immune cell systems might be inevitable. Our results further demonstrated that the flavonoid inhibited HL-60 cell growth via induction of programmed cell death at relatively high concentrations, possibly through activation of $Ca^{2+}$- and $Mg^{2+}$-dependent endonuclease. The tangeretin effects might require *de novo* protein synthesis through progression of the apoptotic pathway. Among the various flavonoids previously examined (Hirano *et al.*, 1994), tangeretin induced apoptosis in HL-60 cells most efficiently, while other flavonoids including hydroxyflavones, mono methoxyflavones, isoflavones, and hydroxychromones did not induce apoptosis in these cells in our assay system (data not shown).

HL-60 cells have been derived from peripheral blood leukocytes of an adult female with acute promyelocytic leukaemia (Collins *et al.*, 1977). Although this cell line is not representative of either lymphoid or epithelial malignancy, and therefore the significance of the data may be limited to this atypical cell line at present, HL-60 has widely been used

**Figure 11** Attenuation by cycloheximide of growth-inhibiting effects of tangeretin. HL-60 cells were treated with 0.0027–27 μM tangeretin in the presence (●) or absence (○) of 5 ng $ml^{-1}$ cycloheximide for 96 h. Then the growth of the cells was determined by an MTT assay method. The percentages of cell growth as compared with controls (without tangeretin) were plotted. The results were shown as mean ± s.e. of triplicate experiments ($n = 3$). Statistically significant effects were observed at tangeretin concentrations of 0.27–27 μM (*$P < 0.01$).

for studies of anti-leukaemic agents or mechanisms of apoptosis (Imaizumi and Breitman, 1987; Boise *et al.*, 1992; Hotz *et al.*, 1992; Gorczyca *et al.*, 1993; Solary *et al.*, 1993; Traganos *et al.*, 1993; Jarvis *et al.*, 1994; Li *et al.*, 1994) for the reason that HL-60 cells easily undergo apoptosis in response to several stimuli (Hotz *et al.*, 1992; Gorczyca *et al.*, 1993; Traganos *et al.*, 1993; Jarvis *et al.*, 1994; Li *et al.*, 1994). Thus, our present results can be discussed with reference to these previous observations, and the data also suggest that it would be worthwhile to further elucidate this novel flavonoid as a candidate for a non-toxic antileukaemia agent using other types of myeloid leukaemia cell lines.

Cytostatic effects of tangeretin on HL-60 cells appeared at relatively low concentrations (<0.27 μM), whereas induction of apoptosis as assessed by morphological changes and DNA fragmentation became apparent when the cells were exposed to 2.7 μM or more tangeretin. These observations suggest that suppression of cell proliferation by tangeretin may proceed before activation of endonuclease and DNA fragmentation. Indeed tangeretin at 27 μM blocked cell proliferation by 58.7% at 24 h culture (data not shown), while the flavonoid at 27 μM induced apoptosis in only 10% of HL-60 cells at this time point. It is possible, however, that cytostasis and apoptosis by tangeretin may occur side by side at relatively high concentrations of tangeretin. The examples of apoptosis known to be induced in mammalian glands, tissues or cells (Bursch, 1990) show that many terminally differentiated cells undergo apoptosis. In most of these cases, the cells that undergo apoptosis are not actively proliferating and are in the $G_0$ or $G_1$ phases of the cell cycle. HL-60 cells have been reported to be able to differentiate into granulocytes or monocytes in response to several stimuli (Samid *et al.*, 1992). Although no apparent evidence for differentiation-inducing effects of tangeretin on HL-60 cells could be obtained under the present experimental conditions, the deficit in the proportion of unaffected cells during the $G_1$ phase coinciding with the appearance of apoptotic cells in cultures treated with tangeretin suggests that $G_1$ phase cells were preferentially undergoing apoptosis in these cultures. Flow cytometric analysis of the treated cells also showed a possibility that the flavonoid may partially perturb cell progression through S and/or $G_2$/M stage and may cause real increases of cells in these phases of cell cycle. It is not clear from the present data of flow cytometric analysis that the cell populations with DNA contents less than 2N (sub $G_1$ populations) denote apoptotic cells. However, the percentage of apoptotic cells calculated from the data from flow cytometric analysis was well correlated with the percentage of such cells calculated from the data from morphological observations (Figure 3), supporting the hypothesis that the sub $G_1$ populations are apoptotic cells. Some recent reports have also considered these sub $G_1$ populations as apoptotic cells (Hotz *et al.*, 1992; Gorczyca *et al.*, 1993; Traganos *et al.*, 1993). In contrast to HL-60 cells, human T-lymphocytic leukaemia MOLT-4 cells or normal human PBMCs hardly responded to both the cytostatic and apoptosis-inducing actions of tangeretin, suggesting that the effect of the flavonoid on HL-60 cells is modulated by cell (tissue) type-specific factors. Data of experiments of long-term treatment with tangeretin or reseeding experiments in drug-free media on HL-60 and MOLT-4 cells treated with tangeretin (Figure 5) support that MOLT-4 cells have a real survival advantage over HL-60 cells.

The time lag for apoptosis induction may depend upon responding cell species or upon various stimuli. For instance, apoptosis of rat fibroblast cell lines was detectable after only 4 h of treatment with cycloheximide, whereas other treatments inducing apoptosis through growth arrest required 24–48 h (Evan *et al.*, 1992). Induction or promotion of apoptosis in human promonocytic U937 cells by interleukin 6 has also been reported to require a time lag of 24–48 h (Afford *et al.*, 1992). In our present studies, apparent changes in apoptosis of HL-60 cells were observed only after 24 h in culture with tangeretin, whereas actinomycin D caused apoptosis in these cells within 6 h in culture (data not shown). Thus, tangeretin appears to require over 24 h to be effective in inducing apoptosis in HL-60 cells. In addition to the time lag effect of tangeretin, the effect was only apparent in a relatively small proportion of the cells in culture (maximum 23%). In general, the apoptotic process takes approximately 3 h to complete (Bursch, 1990). Since the induction of apoptosis is not synchronous throughout the culture, cells at different stages of apoptosis might co-exist. The overall rate of cellular destruction might be very rapid, therefore, it is quite possible that only a minimal percentage of the cells undergoing apoptosis could be observed at any one time during the culture period as in the case of our present experiments. In our assay system, actinomycin D induced DNA fragmentation of HL-60 cells at 0.27 μM, the effect of which was much stronger than that of tangeretin. Actinomycin D at this concentration, however, alternatively induced necrotic cell death of HL-60 cells, whereas tangeretin even at high concentrations (27 μM) did not induce characteristic features of necrotic death, as assessed by morphology and dye exclusion tests.

The activation of an endogenous $Ca^{2+}$- and $Mg^{2+}$- dependent endonuclease is considered to occur in virtually all systems in which apoptosis has been identified (Cohen and Duke, 1984). Although the activation of the endonuclease activity does not require gene transcription or protein synthesis (Bursch, 1990), *de novo* synthesis or activation of many other proteins including transglutaminase (Fesus *et al.*, 1987), TRPM-2 (Leger *et al.*, 1987) and a number of proteinases appear to be required to complete apoptosis. Consistent with these biochemical features of apoptosis, our results strongly suggested that both $Ca^{2+}$- and $Mg^{2+}$- dependent endonuclease activation and *de novo* protein synthesis are critical for tangeretin-induced apoptosis in HL-60 cells. The observations of Cohen and Duke (1984) in a system of thymocyte death by glucocorticoid suggest that the protein for which synthesis is required for cell death is part of a cytoplasm-to-nucleus calcium transport system, and activation of the endonuclease may be the final common pathway in many types of apoptosis.

Tangeretin is known to occur in tangerine peel (Nelson, 1934), which is extensively used in Japan in Kampo medicines for treatment of cancer patients. Our preliminary HPLC data suggest that those Kampo medicines contain some polymethoxyflavonoids including tangeretin in amounts of 100–200 μg $g^{-1}$ of dried extracts of mixed herbs containing tangerine peel. The mechanisms for flavonoid effects on cancer cells in general have not been clarified so far, while inhibition of the membrane sodium pump (Kuriki and Racker, 1976), induction of extensive single-strand breakage (Bissery *et al.*, 1988), inhibition of tyrosine kinase (Akiyama *et al.*, 1987) and blocks of cell cycle progression (Matsukawa *et al.*, 1993) have been considered as possibilities. Inhibition of carcinogenesis (Verma *et al.*, 1988) and angiogenesis (Fotsis *et al.*, 1993) are other aspects of flavonoid efficacy for cancer treatment. From these observations, the anti-cancer activity of the flavonoids appears not to be due to a common mechanism. In addition to these flavonoid actions, a recent report by Yanagihara *et al.* (1993) suggested that isoflavones genistein and biochanin A, contained in soy bean diets, suppressed both *in vitro* and *in vivo* growth of human cancer cell lines established from the gastrointestinal tract, possibly via induction of apoptosis in these cells. These observations, including our present findings of tangeretin effects, suggest that at least some flavonoids of diet origin may play an important role in the regulation of programmed malignant cell death. However, the activity of flavonoids in inducing apoptosis of malignant cells appears to be limited to certain types of the compounds, such as isoflavones or polymethoxyflavones.

The present study, in summary, proposes for the first time, a growth-inhibitory effect of a citrus polymethoxyflavone tangeretin against HL-60 cells at least partially through induction of programmed cell death with less cytotoxicity on normal human PBMCs. In the process of tangeretin-induced apoptosis, activation of $Ca^{2+}$- and $Mg^{2+}$-dependent endonuclease and *de novo* protein synthesis might be required.

**Acknowledgements**

This study was supported by grant from the Ministry of Education of Japan (grant number 06672279) as well as grant of Kanagawa Academy of Science and Technology (grant number 94042).

**References**

ADLERCREUTZ H. (1984). Does fiber-rich food containing animal lignan precursors protect against both colon and breast cancer? An extension of the fiber hypothesis. *Gastroenterology*, **86**, 761–766.

ADLERCREUTZ H, FOTSIS T, HEIKKINEN R, DWYER JT, WOODS M, GOLDIN BR AND GORBACH SL. (1982). Excretion of the lignans enterolactone and enterodiol and of equol in omnivorous and vegetarian postmenopausal women and in women with breast cancer. *Lancet*, **2**, 1295–1299.

AFFORD SC, PONGRACZ J, STOCKLEY RA, CROCKER J AND BURNETT D. (1992). The induction by human interleukin-6 of apoptosis in the promonocytic cell line U937 and human neutrophils. *J. Biol. Chem.*, **267**, 21612–21616.

AKIYAMA T, ISHIDA J, NAKAGAWA S, OGAWA H, WATANABE S, ITOH N, SHIBUYA M AND FUKAMI Y. (1987). Genistein, a specific inhibitor of tyrosine-specific protein kinase. *J. Biol. Chem.*, **262**, 5592–5595.

ARENDS MJ, MORRIS RG AND WYLLIE AH. (1990). Apoptosis: the role of endonuclease. *Am. J. Pathol.*, **136**, 593–608.

BARRY MA, BEHNKE CA AND EASTMAN A. (1990). Activation of programmed cell death (apoptosis) by cisplatin, other anticancer drugs, toxins and hyperthermia. *Biochem. Pharmacol.*, **40**, 2353–2362.

BERTRAND R, KERRIGAN D, SERANG M AND POMMIER Y. (1991). Cell death induced by topoisomerase inhibitors. *Biochem. Pharmacol.*, **42**, 77–85.

BISSERY M-C, VALERIOTE FA, CHABOT GG, CRISSMAN JD, YOST C AND CORBELT TH. (1988). Flavone acetic acid (NSC 34 7512)-induced DNA damage in glasgow osteogenic sarcoma in vivo. *Cancer Res.*, **48**, 1279–1285.

BOISE LH, GRANT S AND WESTIN EH. (1992). Altered expression of c-myb in a subclone of HL-60 that exhibits reversible differentiation. *Cell Growth Differ*, **3**, 53–61.

BURSCH W. (1990). The biochemistry of cell death by apoptosis. *Biochem. Cell Biol.*, **68**, 1071–1074.

CHANG MP, BALDWIN RL, BRUCE C AND WISNIESKI BJ. (1989). Second cytotoxic pathway of diphtheria toxin suggested by nuclease activity. *Science*, **246**, 1165–1168.

COLLINS SJ, GALLO RC AND GALLAGHER RE. (1977). Continuous growth and differentiation of human myeloid leukaemic cells in suspension culture. *Nature*, **270**, 347–349.

COHEN JJ AND DUKE RC. (1984). Glucocorticoid activation of a calcium-dependent endonuclease in thymocyte nuclei leads to cell death. *J. Immunol.*, **132**, 38–42.

COMPTON MM. (1992). A biochemical hallmark of apoptosis: internucleosomal degradation of the genome. *Cancer Metastasis Rev.*, **11**, 105–119.

DIVE C AND HICKMANN JA. (1991). Drug-target interactions: only the first step in the commitment to a programmed cell death? *Br. J. Cancer*, **64**, 192–196.

DUKE RC, CHERVENAK R AND COHEN JJ. (1983). Endogenous endonuclease-induced DNA fragmentation: an early event in cell-mediated cytolysis. *Proc. Natl Acad. Sci.*, **80**, 6361–6365.

EDWARDS JM. (1979). Antineoplastic activity and cytotoxicity of flavone, isoflavone, and flavanones. *J. Natural Product*, **42**, 85–91.

EVAN GI, WYLLIE AH, GILBERT CS, LITTLEWOOD TD, LAND H, BROOKS M, WATERS CM, PENN LZ AND HANCOCK DC. (1992). Induction of apoptosis in fibroblasts by c-myc protein. *Cell*, **69**, 119–128.

FESUS L, THOMAZY V AND FALUS A. (1987). Induction and activation of tissue transglutaminase during programmed cell death. *FEBS Lett.*, **224**, 104–108.

FOTSIS T, PEPPER M, ADLERCREUTZ H, FLEISCHMANN G, HASE T, MONTESANO R AND SCHWEIGERER L. (1993). Genistein, a dietary-derived inhibitor of in vitro angiogenesis. *Proc. Natl Acad. Sci.*, **90**, 2690–2694.

GORCZYCA W, GONG J, ARDELT B, TRAGANOS F AND DARZYNKIEWICZ Z. (1993). The cell cycle related differences in sensitivity of HL-60 cells to apoptosis induced by various antitumor agents. *Cancer Res.*, **53**, 3186–3192.

HICKMAN JA. (1992). Apoptosis induced by anticancer drugs. *Cancer Metastasis Rev.*, **11**, 121–139.

HIRANO T, OKA K AND AKIBA M. (1989*a*). Antiproliferative effects of synthetic and naturally occurring flavonoids on tumor cells of the human breast carcinoma cell line, ZR-75-1. *Res. Commun. Chem. Pathol. Pharmacol.*, **64**, 69–78.

HIRANO T, OKA K, KAWASHIMA E AND AKIBA M. (1989*b*). Effects of synthetic and naturally occurring flavonoids on mitogen-induced proliferation of human peripheral-blood lymphocytes. *Life Sci.*, **45**, 1407–1411.

HIRANO T, FUKUOKA K, OKA K, NAITO T, HOSAKA K, MITSUHASHI H AND MATSUMOTO Y. (1990). Antiproliferative activity of mammalian lignan derivatives against human breast carcinoma cell line, ZR-75-1. *Cancer Inv.*, **8**, 595–602.

HIRANO T, GOTOH M AND OKA K. (1994). Natural flavonoids and lignans are potent cytostatic agents against human leukemic HL-60 cells. *Life Sci.*, **55**, 1061–1069.

HOTZ MA, DEL BINO G, LASSOTA P, TRAGANOS F AND DARZYNKIEWICZ Z. (1992). Cytostatic and cytotoxic effects of fostriecin on human promyelocytic HL-60 and lymphocytic MOLT-4 cells. *Cancer Res.*, **52**, 1530–1535.

IMAIZUMI M AND BREITMAN TR. (1987). Retinoic acid-induced differentiation of the human promyelocytic leukemia cell line, HL-60, and fresh human leukemia cells in primary culture: a model for differentiation inducing therapy of leukemia. *Eur. J. Haematol.*, **38**, 289–302.

JARVIS WD, TURNER AJ, POVIRK LF, TRAYLOR RS AND GRANT S. (1994). Induction of apoptotic DNA fragmentation and cell death in HL-60 human promyelocytic leukemia cells by pharmacological inhibitors of protein kinase C. *Cancer Res*, **54**, 1707–1714.

KURIKI Y AND RACKER E. (1976). Inhibition of ($Na^+$, $K^+$) adenosine triphosphatase and its partial reactions by quercetin. *Biochemistry*, **15**, 4951–4956.

LEGER LG, MONTPETIT ML AND TENNISWOOD MP. (1987). Characterization and cloning of androgen-repressed mRNAs from rat ventral prostate. *Biochem. Biophys. Res. Commun.*, **147**, 196–203.

LI X, TRAGANOS F AND DARZYNKIEWICZ Z. (1994). Simultaneous analysis of DNA replication and apoptosis during treatment of HL-60 cells with camptothecin and hyperthermia and mitogen stimulated human lymphocytes. *Cancer Res*, **54**, 4289–4293.

MATSUKAWA Y, MATUI N, SAKAI T, SATOMI Y, YOSHIDA M, MATSUMOTO K, NISHINO H AND AOIKE A. (1993). Genistein arrest cell cycle progression at $G_2$-M. *Cancer Res.*, **53,** 1328–1331.

MISHELL BB, SHIIGI SM, HENRY C, CHAN EL, NORTH J, GALLILY R, SLOMICH M, MILLER K, MARBROOK J, PARKS D AND GOOD H. (1980). Preparation of mouse cell suspensions. In *Selected Methods in Cellular Immunology*, Mishell BB and Shiigi SM. (eds) pp. 3–27. W.H. Freeman and Company: San Francisco.

NELSON EK. (1934). The occurrence of pentamethyl flavonol in tangerine peel. *J. Am. Chem. Soc.*, **56,** 1392–1393.

NICOLAOU KC, STABILA P, ESMAELI-AZAD B, WRASIDLO W AND HIATT A. (1993). Cell-specific regulation of apoptosis by designed enediynes. *Proc. Natl Acad. Sci.*, **90,** 3142–3146.

SAMID D, SHACK S AND SHERMAN LT. (1992). Phenylacetate: a novel nontoxic inducer of tumor cell differentiation. *Cancer Res.*, **52,** 1988–1992.

SARGENT JM AND TAYLOR CG. (1989). Appraisal of the MTT assay as a rapid test of chemosensitivity in acute myeloid leukaemia. *Br. J. Cancer*, **60,** 206–210.

SERVOMAA K AND RYTOMAA. (1990). UV light and ionizing radiations cause programmed cell death of rat chloroleukaemia cells by inducing retropositions of a mobile DNA element (L1Rn). *Int. J. Radiat. Biol.*, **57,** 331–343.

SETCHELL KDR, LAWSON AM, BORRIELLO SP, HARKNESS R, GORDON H, MORGAN DML, KIRK DN, ADLERCREUTZ H, ANDERSON LC AND AXELSON M. (1981). Lignan formation in man: microbial involvement and possible roles in relation to cancer. *Lancet*, **1,** 4–7.

SOLARY E, BETRAND R, KOHN KW AND POMMIER Y. (1993). Differential induction of apoptosis in undifferentiated HL-60 cells by DNA topoisomerases I and II inhibitors. *Blood*, **81,** 1359–1368.

SUOLINNA E-M, BUCHSBAUM RN AND RACKER E. (1975). The effect of flavonoids on aerobic glycolysis and growth of tumor cells. *Cancer Res.*, **35,** 1865–1872.

TRAGANOS F, KAPUSCINSKI J, GONG J, ARDELT B, DARZYNKIEWICZ RJ AND DARZYNKIEWICZ Z. (1993). Caffein prevents apoptosis and cell cycle effects induced by camptothecin or topotecan in HL-60 cells. *Cancer Res.*, **53,** 4613–4618.

VERMA AK, JOHNSON JA, GOULD MN AND TANNER MA. (1988). Inhibition of 7,12-dimethylbenz(*a*)anthracene- and *N*-nitrosomethylurea-induced rat mammary cancer by dietary flavonol quercetin. *Cancer Res.*, **48,** 5754–5758.

WRIGHT SC, KUMAR P, TAM AW, SHEN N, VARMA M AND LARRICK JW. (1992). Apoptosis and DNA fragmentation precede TNF-induced cytolysis in U937 cels. *J. Cellular Biochem.*, **48,** 344–355.

WYLLIE AH. (1985). The biology of cell death in tumors. *Anticancer Res.*, **5,** 131–142.

WYLLIE AH, KERR JF AND CURRIE AR. (1980). Cell death: the significance of apoptosis. *Int. Rev. Cytol.*, **68,** 251–306.

YANAGIHARA K, ITO A, TOGE T AND NUMOTO M. (1993). Antiproliferative effects of isoflavones on human cancer cell lines established from the gastrointestinal tract. *Cancer Res.*, **53,** 5815–5821.